# Research with pregnant women: a call to action

**DOI:** 10.1186/s12978-017-0419-x

**Published:** 2017-12-14

**Authors:** Margaret Olivia Little, Marisha N. Wickremsinhe

**Affiliations:** Kennedy Institute of Ethics, Georgetown University, Washington, DC USA

**Keywords:** Pregnant women, Clinical research, Ethics

## Abstract

Despite a global need for the use of medication during pregnancy, the medical research community lacks robust evidence for safety and efficacy of treatments and preventives often taken by pregnant women. Given the biological differences between pregnant women and the rest of the population, the need to gather data on the ways in which medications behave in the pregnant body is critical to the health of pregnant women and their offspring. Three ethical reasons are central to this need: 1. Pregnant women deserve access to *effective* treatment, 2. Pregnant women deserve access to *safe* treatment, and 3. Pregnant women deserve *equitable access* to trials carrying the prospect of direct benefit. In this paper, we introduce and frame this Supplement Issue, which presents important conference proceedings of the 2016 Global Forum on Bioethics in Research meeting held in Buenos Aires, Argentina, on the 3rd and 4th of November.

## The need to pursue research with pregnant women

Globally, a significant proportion of pregnancies are complicated by serious medical illness requiring treatment [1]. Despite an assumption—or perhaps a wish—that pregnancy occurs only against a backdrop of “perfect health,” women often confront illness in the face of pregnancy or pregnancy in the face of illness. A multitude of examples come to mind: a pregnant woman contracts malaria, a woman with diabetes becomes pregnant, a woman with a persistent psychiatric illness that is well-controlled by medication wants to have a child, or perhaps a pregnant woman is diagnosed with HIV at her first antenatal care visit. In each of these cases, the necessity of medication use during pregnancy to treat or manage chronic disease or new infection is unquestionable.

In fact, a significant proportion of pregnant women take medication at some point during their pregnancy, if not throughout the course of their pregnancy. In the United States, as many as 70% of women took at least one prescription medication during pregnancy [2]. Certain disease states make this need vivid. For example, in the global context of HIV/AIDS, medication use during pregnancy is essential: approximately 1.5 million women living with HIV give birth across the world each year [3] and use antiretroviral medicines to manage their illness and prevent mother-to-child transmission of the infection.

To understand the significance of the burden of disease and subsequent use of medications during pregnancy, however, we must acknowledge that medications behave differently in the pregnant body [4]. Just as treating children as merely miniature adults in the context of medical science is irresponsible, so, too, is treating pregnant women as merely women with big bellies [1]. The pregnant body can act as a “wild card” when it comes to metabolizing medications—during pregnancy, certain medications dosed for non-pregnant adults can clear the body too quickly to offer therapeutic benefit [4]. Others may specifically target the developing fetus, causing harm, and fail to treat the pregnant woman’s illness at all.

But we lack robust evidence to assess the ways in which medications are metabolized by the pregnant body, the extent to which medications treat the woman’s health condition, and the degree to which medications affect the fetus. Indeed, pregnant women have been called the “last true therapeutic orphan” [5]. Against this backdrop, the reasons to pursue research with pregnant women are not just medical, they are ethical.

## Three ethical reasons for pursuing research with pregnant women

There are three central ethical reasons to pursue research with pregnant women: 1. Pregnant women deserve access to *effective* treatment, 2. Pregnant women deserve access to *safe* treatment, and 3. Pregnant women deserve *equitable access* to treatment.

Firstly, pregnant women deserve access to *effective* treatments and preventives [6]. Failure to properly collect evidence in the clinical research setting leads to uncertainty in the clinical care setting. Without robust knowledge of medication efficacy in pregnancy, pregnant women and their providers are forced to make decisions on the basis of substandard data. In some cases, treatment regimens are determined by data collected from extensive registries following incidental exposure; in other cases, treatment regimens are determined by past anecdotal evidence. Treating without more complete evidence can lead to sub-therapeutic dosing of medications in pregnant women, wherein the disease progresses unchecked because the pregnant body metabolizes the compound too quickly. Clearly, pregnant women deserve medical care to effectively treat their medical conditions, and research is needed to ensure this.

Secondly, pregnant women and the offspring they carry deserve access to *safe* treatments and preventives [6]. The definitive case to exemplify the consequences of failing to conduct fetal safety research, well-known to most, is thalidomide. Improperly researched, thalidomide was used widely in pregnancy without knowledge of its detrimental effects on the fetus [7]. Without proper data, we not only risk failing to effectively treat the pregnant woman’s illness—we risk failing to protect the fetus from harm. At present, the lack of research in pregnancy results in a shift of risk from the controlled, highly regulated context of a research trial to the unmonitored, highly variable context of the clinic [8]. Such a transfer of risk ultimately jeopardizes fetal safety for a far greater proportion of pregnancies than does conducting necessary research in the context of a small and rigorously monitored safety trial.

Thirdly, pregnant women deserve *equitable access* to research trials that carry the “prospect of direct benefit”, i.e. later-stage trials that involve a potential for therapeutic benefit to participants. Depending on the disease state in question, such trials can be important sources of medical benefit, and sometimes—as in emergency situations—the only source. Yet pregnant women are often summarily excluded, without justification, from such research [6]. This exclusion prevents pregnant women and their offspring from accessing an intervention that may be critical for their health. Not only should we provide effective treatment to women, and not only should we adequately assesses fetal safety of medications; we should ensure as a matter of justice that pregnant women are offered equitable opportunity to participate in such trials [6].

## The way forward

Research with pregnant women certainly carries special moral complexities. Such research, after all, involves two intertwined entities—the woman, who can consent to the risks of research, and the future offspring she carries, which cannot. The importance of establishing independent protections for that future offspring is a consensus issue [8]. The vast majority of research that can (and should) include pregnant women involves pregnancies that continue to term, and that hence would implicate a future child whose health, all agree, is important. That said, these protections, centered around the presence of the fetus, cannot displace the importance of the pregnant woman’s specific health needs. Women do not forfeit all rights of access to important medical benefit just because they are continuing a pregnancy [6]. Clearly, then, research with pregnant women requires both protecting the future child while respecting the pregnant woman as autonomous in her own right.

Increasingly, key organizations—including the Pan American Health Organization, Council for International Organizations of Medical Sciences, American College of Obstetrics and Gynecology, and the Office of Research on Women’s Health of the National Institutes of Health—have recognized the scientific and ethical importance of including pregnant women in clinical research [9–12]. These organizations encourage a paradigm shift from categorical exclusion to equitable inclusion in the research agenda, while also acknowledging that research with pregnant women poses unique ethical complexities because of risks and potential benefits to future offspring who cannot consent for themselves. They urge a shift in mindset from protecting pregnant women and their future offspring not *from* research, but *through* research.

We must motivate a collective move from the presumption of exclusion to the presumption of inclusion in the research agenda [4]. We should ask not “What is the risk of doing research with pregnant women?”, but “What is the risk of *not* doing this research?”. The current culture of exclusion, fed by misunderstandings and myths, remains a critical barrier to the equitable inclusion of pregnant women in research, not only to address the woman’s own health needs or to ensure the safety of the fetus, but as a matter of justice. Justice demands that we find the moral courage to commit to the very premise of clinical research itself: to seek robust evidence in the face of uncertainty, rather than forcing the public and their healthcare providers to guess.

## Galvanizing the research community

In this Supplement Issue, we aim to help galvanize the research community to take up this critical issue and work toward the ethical inclusion of pregnant women in the research agenda. The articles in this Supplement are adapted from presentations given at the 2016 Global Forum on Bioethics in Research (GFBR) meeting, “Ethics of research in pregnancy”, which took place in Buenos Aires, Argentina, on the 3rd and 4th of November. The GFBR, supported by the Wellcome Trust, the Bill & Melinda Gates Foundation, the National Institutes of Health, and the UK Medical Research Council, hosts annual meetings on key bioethics topics.

The issue opens with two foundational pieces. The first outlines the global burden of disease in pregnancy, originally a background report for conference participants; the second explores the regulatory landscape of research with pregnant women. The issue then moves to series of eight case studies that exemplify ethical challenges of including pregnant women in research, drawing from a diversity of countries, disease states, and study designs. The first two focus on diabetes-related interventions. They are followed by two cases that concern the use of pre-exposure prophylaxis for HIV during pregnancy. The next three case studies look at issues specific to research on obstetric conditions, and the last is a case centered around one pregnant woman during the Ebola epidemic, highlighting the very real, very human costs of the exclusion of pregnant women from research. A longer form article then provides a deep dive, from the perspective of the Ethics Review Committee of the World Health Organization, into the systematic exclusion of pregnant women from clinical trials during the Ebola epidemic. The final article offers a brief summary of the 2016 GFBR conference.

Ultimately, we hope this Supplement Issue presents a “call to action”: we ask the international research community to ensure, as a matter of social justice, that pregnant women are responsibly, ethically, and equitably included in clinical research endeavors moving forward.

## Abbreviations

GFBR: Global Forum on Research in Bioethics
